# Empyemas of the Accessory Cavities of the Nose in Children1Presented at the meeting of the Roswell Park Medical Club, at the residence of Dr. Geo. F. Cott, August, 1904.

**Published:** 1905-02

**Authors:** B. Panzer

**Affiliations:** Vienna University Children’s Hospital


### Empyemas of the Accessory Cavities of the Nose in Children.[1]

By DR. B. PANZER,
Of Vienna University Children's Hospital.

I WISH to speak to you about the special form of a disease which to state beforehand must be considered very rare. I shall speak about empyemas of the accessory cavities of the nose as found with children. I did not find more than seven cases in a material of about fifteen thousand subjects that came under my observation within the last ten years. Nevertheless, the cases presented typical forms which showed marked differences, however, when compared with similar diseases in adults. This latter affection may be taken as very frequent, for I have seen many hundreds of such cases during the same time. As the disease represented always the same well-known symptoms as regards its anatomical, pathological, and therapeutical features, it might be well to consider first the affection in adults as, by comparison, we shall be able to recognise best the special points of same.

I will recapitulate briefly the anatomy of the accessory cavities of the nose: the Highmore cavity, the frontal sinus, ethmoidal labyrinth, and the sphenoidal cavity. The antrum of Highmore, the largest of all, is situated in the upper jaw, is an irregular cavity, narrowing down toward the alveolar process. The exit is close to the roof which forms at the same time the base of the orbit. Later, I shall refer to the openings in the nose. The frontal sinus varies very greatly in its size, lies inward and upward of the orbit, but extends also outward as mentioned before, in

1. Presented at the meeting of the Roswell Park Medical Club, at the residence of Dr. Geo. F. Cott, August, 1904.

varying sizes. The duct leads obliquely from upward behind, to downward in front. The ethmoidal labyrinth is divided into two parts, the anterior and the posterior cells; they represent a number of irregular cavities which are partly in communication and are limited on the outer side by the lachrymal bone and the lamina papyracea of the ethmoid, in the interior by the upper turbinated bone. The sphenoidal cavity lies in the body of the sphenoid bone, and is a large, more or less, cubical space limited on all sides by bone; in front especially by the so-called ossicula Bertini.

Of great importance is the relation of the openings to the nose. Make a sagittal section through the skull, remove in the middle the septum and you will notice in the lower part the inferior turbinated bone and between this and the floor of the nose the lower meatus, over it the middle turbinated bone and between this and the lower turbinated bone the middle meatus; above this the upper turbinated bone and a portion of the anterior ethmoidal labyrinth. Remove now the middle turbinated bone and you will find two well organised formations on the outer side of the nasal wall,—in front, the processus uncinatus of the ethmoid, and behind this, the ethmoidal bulla. These two bones have special importance, for between these two there is a furrow,—the so-called hiatus semilunaris, on the upper end of which there is the nasofrontal duct, the opening of the frontal sinus. In the lower part of the ostium maxillare of the Highmore cavity further there are the openings of some ethmoid cells next to the hiatus, in irregularly situated parts; whereas the posterior ethmoidal cells open into the back parts of the upper nasal meatus. In the same way the opening of the anterior wall of the sphenoidal cavity leads into the upper meatus.

Let us contemplate now the sagittal section of the skull with the middle turbinated bone in the natural position, when we shall see that the Highmore cavity, the frontal sinus and the anterior cells of the ethmoid open into the middle meatus. The narrow fissure which we observe in examination of the nose from the front, between the lower and the middle turbinated bone, possesses the greatest practical importance. Whatever occurs in any of the said cavities must show up by indication in this fissure, as we cannot get a direct view of any of these cavities, whether of the Highmore cavity of the frontal sinus or of the ethmoid, but you will always find a strip of pus in the fissure between the lower and the middle turbinated bone, as the first symptom in examining the nose. The question arises how to make the differential diagnosis of the disease of the different cavities. The simplest way is to ask, which one is most frequent? The comparison shows that in the great majority of cases the affection lies in the Highmore cavity; finding some pus in the middle meatus, you must imagine first an inflammation of this cavity.

The diagnosis is very easily verified. Remembering the anatomy, you know that the bottom of the Highmore cavity reaches to the alveolar process, consequently is somewhat deeper than the bottom of the nasal cavity. Take now a simple hollow needle and, under local anesthesia, introduce it underneath the lower turbinated bone about one-half inch to the back from the anterior end of the lower turbinated bone, right through the lateral wall of the nasal cavity which is at the same time the medial wall of the Highmore cavity, and you will reach with your instrument the Highmore cavity. Take now a syringe with warm water and adding 5 per cent. carbolic, and press it through the cavity; the fluid must come out through the natural opening and we are able to distinguish whether there is any pus in the cavity or not; when the water shows any pus you can be sure that there is an empyema of the Highmore cavity. When the fluid is clear the Highmore cavity is free of any affection, and it is only possible that there is an empyema of the frontal sinus or of the ethmoid.

The entrance to the frontal sinus is easily to be found: take a curved canula, introduce it below the anterior end of the middle turbinated bone and you will reach through the hiatus semilunaris into the end of the nasofrontal duct. Sometimes the anterior end of the middle turbinated bone is in the way, in which case you must remove it with the snare; and now you repeat the former proceeding, that is, you syringe some water through the tube; if you find any pus in the fluid you can be sure there is a frontal empyema; if you do not find any pus it cannot be but an empyema of the ethmoid.

But how will the case present itself if there should be a combined empyema in several of the above mentioned cavities? The diagnosis could not be difficult in such a case either; should there be visible pus in the middle meatus after thorough washing out of the Highmore cavity, this is a clear indication that there is still suppuration either in the frontal sinus or in the ethmoid. If after washing out the frontal sinus there is still pus, the ethmoid must be affected. The general symptoms of the empyema visible besides the examination of the nose,—and I am speaking here only of the acute form of empyema,—are in the first line pains, pains of a neuralgic character which mostly, but not always, correspond to the seat of the trouble, pains in the infraorbital nerve in disease of the Highmore cavity, in the supraorbital nerve in those of the head. But also radiating pains of an affection of the Highmore cavity in the forehead. The region of the infraorbital foramen and supraorbital fissure are sensitive against pressure, redness of the conjunctivæ half closing of the eye are not very frequent. I repeat, the pains are of a neuralgic character, that is, they return often at the same hour, or the branches of the trigeminus nerve show a general sensitiveness and the pains become stronger at certain hours. Fever is not frequent, but slight increases of the temperature can often be observed. On the whole, these are the usual signs in adults, but we find them quite different with children, of course, to state it at once, the symptoms, which we may call the anatomical are always the same.

The suppuration in any of the cavities will always show up in the nose in the same place. Also, we always find pus in the middle meatus between the lower and the middle turbinated bone in case of an affection of the frontal sinus or Highmore cavity. But the exterior appearances are quite different. The empyema manifests itself under the signs of a serious general illness, high fever as much as 40° C. and more, sometimes somnolence and dulness of the intellect. The most pregnant symptoms are the pains which appear spontaneously and mostly with enormous intensity, very much stronger than ever in adults. The branches of the trigeminus are extremely sensitive against pressure. Examining the patient you cannot but notice immediately the severe edema which corresponds to the part of the affected cavity. In case of the inflammation of the Highmore cavity severe edema of the cheek, swelling of the eyelids strong injection and swelling of the conjunctivæ which can reach the state of chemosis. In case of the affection of the frontal sinus you will find the edema above the eyebrows on the forehead as far as the middle line and beyond same, and even so far as the other side. The conjunctiva is affected in the same way as in case of an inflammation of the Highmore cavity. On examination of the nose there is to be noticed a large quantity of mucopurulent secretion in the middle meatus, exactly as we used to find it with adults. The most important differences, however, appear in its course. Same becomes relatively as mild as the beginning was strong and sudden. In no case have I seen the disease changed into a chronic one and in all cases I found the conservative treatment quite sufficient. In cases of adults it will rarely be possible to heal the suppuration, for example of the Highmore cavity, without any washing out of the cavity. I never found this necessary with children, nevertheless the treatment can do a great deal and I found the following way the best: it is most necessary to procure free egress for the pus; as a swelling of the mucous membrane of the middle turbinated bone occurs always in this disease, it must be our task to reduce this swelling as much as possible.

This result is easiest obtained by use of cocaine and, for the past two years, adrenalin. I use a 20 per cent. solution of cocaine in the usual solution of adrenalin, 1-10, and introduce a probe with cotton wetted therewith into the middle meatus outward of the middle turbinated bone. Within a few minutes the mucous membrane contracts, becomes anemic and often perfectly white, and we may observe a nearly immediate effect,—the diminution of pain,—for the pus can now leave freely. A good result is additionally obtained by giving aspirin in large doses, according to the age of the children, using further a great deal of fluids, especially hot tea, and regulating the diet. The external application of mesotan with oil, equal parts, on the diseased and painful place will be found quieting, combined with the application of ice; but sometimes the little patient cannot stand the cool, and tolerates fomentations better. The fever and pains, as a rule, last but four or five days and mostly disappear, whereas the suppuration usually lasts a fortnight or three weeks. The treatment remains the same as long as we find any pus; the nose might be cautiously washed out to make it permeable, only great care must be taken that the fluid does not penetrate in the Eustachian tube, as this would cause immediately a severe inflammation of the middle ear in consequence of the septic pus. When not absolutely necessary, it is much better to omit the washing out. The following cases are instructive:

CASE I.—A child, 4 years old, comes with its mother to the hospital; the child has high fever, terrible pains, cries all night. Since yesterday there is a swelling on the cheek. The eye is swollen and very red. There are great pains when you touch the swollen part. The examination of the nose shows up yellow pus in the middle meatus.

Diagnosis: acute empyema of the left Highmorean cavity. Therapy as described above. The pains last for three days with great intensity so I thought, to relieve the child, it would be necessary to perform a puncture of the Highmore cavity, but during the night the pains were gone. The skin on the swollen part came off and after a fortnight the process was ended.

CASE II.—A child of 12 years called for consultation. I found high fever, terrible pains, severe edema above the eye, chemosis of the conjunctivæ, sensitiveness against pressure on the supraorbital nerve. The physician thought there might be a retrobulbar phlegmon, but the oculist had found that the eye itself was normal. The examination of the nose showed pus in the middle meatus.

Diagnosis: frontal sinus empyema. The treatment was the same as in the first case.

Case III.—Child, 10 years old, case as the second; especially remarkable by the pains and high fever; dulness of the intellect. Diagnosis: frontal empyema; fever and pains last, but after seven days the process was the same as in the other cases.

Case IV.—Boy, 12 years old, called upon me in the hospital with the diagnosis empyema of the Highmore cavity. The diagnosis was made by another specialist and it was intended to open at once the Highmore cavity from front without trying any local treatment. I started with the same treatment as experienced in the other cases and the farther progress was that the case, which came eight days after beginning of the disease under my observation was slower than I used to see; it took about a week before the acute signs were gone; during the examination the exudate changed from a purulent to a mucopurulent, and lastly into mucus. It took more than five weeks to finish the whole process.

I will not trouble you longer with the description of other cases, as they have nearly the same histories, there being slight differences only in regard to the pains, the fever and the time for cure.

